# Highly selective synthesis of (*E*)-alkenyl-(pentafluorosulfanyl)benzenes through Horner–Wadsworth–Emmons reaction

**DOI:** 10.3762/bjoc.8.131

**Published:** 2012-07-25

**Authors:** George Iakobson, Petr Beier

**Affiliations:** 1 Institute of Organic Chemistry and Biochemistry, Academy of Sciences of the Czech Republic, Flemingovo nám. 2, 166 10 Prague, Czech Republic

**Keywords:** Horner–Wadsworth–Emmons reaction, pentafluorosulfanyl group, phosphonates, sulfurpentafluoride

## Abstract

Diethyl 2-nitro-(pentafluorosulfanyl)benzylphosphonates, available by the vicarious nucleophilic substitution reaction of *meta-* and *para-*nitro-(pentafluorosulfanyl)benzenes and diethyl chloromethylphosphonate, undergo Horner–Wadsworth–Emmons reaction with aldehydes in the presence of potassium hydroxide in acetonitrile at ambient temperature to give (*E*)-2-nitro-1-alkenyl-(pentafluorosulfanyl)benzenes in good yields and high stereoselectivities. Follow-up transformations of the primary products provided (*E*)-1-alkenyl-(pentafluorosulfanyl)benzenes and 2-(2-arylethyl)-(pentafluorosulfanyl)anilines.

## Introduction

Although the pentafluorosulfanyl (SF_5_) containing compounds have been known for more than half a century [1–4], they remain a relatively underdeveloped class of compounds. This is so despite the unusual combination of properties that the SF_5_ group possesses, such as high thermal and chemical stability, high electronegativity and strong lipophilic character [2–7]. The availability of SF_5_-containing compounds is very limited. Aliphatic SF_5_-containing compounds are available through free-radical addition of toxic and expensive SF_5_Cl to unsaturated compounds [8–9], and aromatic *meta*- and *para*-nitro-(pentafluorosufanyl)benzenes (**1** and **2**) are made by direct fluorination of the corresponding bis(nitrophenyl)disulfides [10–12]. A recent report also described a two step synthesis of SF_5_-benzenes from diaryl disulfides avoiding the use of elemental fluorine [13]. While the only known S_E_Ar of **1** or **2** is the nitration of **2** under harsh conditions and in low yield [14], we have recently described S_N_Ar of the nitro group in compounds **1** and **2** with alkoxides and thiolates [15], vicarious nucleophilic substitution (VNS) of the hydrogen with carbon [16], oxygen [17] and nitrogen [18] nucleophiles, and the oxidative nucleophilic substitution with Grignard and alkyllithium reagents [19]. Reduction of the nitro group in **1** or **2** to (pentafluorosulfanyl)anilines followed by acylation, S_E_Ar halogenation or diazotization (with follow-up reactions) has also been described [11,20–22].

**Scheme 1:**
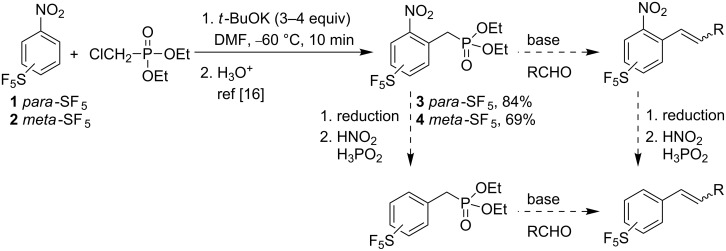
Proposed synthesis of alkenyl-(pentafluorosulfanyl)benzenes.

Alkenyl substituted SF_5_-benzenes or SF_5_-containing stilbene derivatives are not known and would represent basic synthetic intermediates towards more elaborate structures. We envisioned a synthetic route towards these compounds through Horner–Wadsworth–Emmons (HWE) reaction of phosphonates **3** and **4**, which are available by vicarious nucleophilic substitution (VNS) of commercial nitrobenzenes **1** and **2** with diethyl chloromethylphosphonate [16]. If required, the nitro group can be removed by a reduction/diazotization/reduction sequence before or after the HWE reaction (Scheme 1).

The HWE reaction is a modification of the Wittig olefination in which a phosphoryl-stabilized carbanion reacts with an aldehyde or ketone to form an alkene and a water-soluble phosphate ester [23–25]. In general, this reaction preferentially gives more stable *E*-disubstituted alkenes, although several successful attempts have been made to favor *Z*-alkenes [26–28].

## Results and Discussion

The HWE reaction of phosphonate **3** with benzaldehyde in the presence of a base giving stilbene derivative **5a** was investigated. At first, attempts were made to form **5a** directly from **1** and diethyl chloromethylphosphonate by a two-step one-pot process, involving VNS reaction in DMF with a three-fold excess of *t*-BuOK (−60 °C, 10 min), followed by the addition of benzaldehyde (1.5 equiv) and warming of the reaction mixture to 50 °C (Table 1, entry 1). These conditions provided the expected **5a** in good GCMS yield and high *E*/*Z* selectivity, but called for rather long reaction time. By changing the solvent from DMF to THF, we observed a less efficient first step (the VNS reaction) with many unidentified side products being formed (Table 1, entry 2). Therefore, all other experiments were carried out starting from isolated phosphonate **3**. We found that various bases mediate the HWE reaction. *t*-BuOK in THF gave good results; however, the reaction required heating to 50 °C for at least one hour. With *n*-BuLi the reaction is complete at ambient temperature in about half an hour. By using an extended reaction time, much less basic potassium or caesium carbonate in acetonitrile could also be used successfully. Finally, we have identified potassium hydroxide as a very inexpensive and convenient base. The best results were obtained by using 1.8 equiv of KOH in acetonitrile. The addition of small amounts of water increased the reaction rate, presumably by better solubilization of KOH and the formed potassium diethylphosphate, and gave the required product **5a** in 84% isolated yield and high *E*/*Z* selectivity (Table 1, entry 13).

**Table 1:** Optimization of HWE reaction of phosphonate **3** with benzaldehyde.

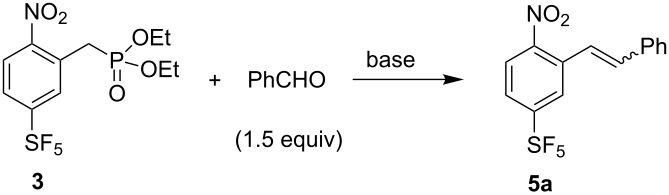						

Entry	Base (equiv)	Solvent	*T* (°C)	*t* (min)	**5a**, Yield (%)^a^	*E*/*Z*^b^

1^c^	*t*-BuOK (3.0)	DMF	50	960	83	97:3
2^c^	*t*-BuOK (3.0)	THF	50	120	58	>98:2
3	*t*-BuOK (3.0)	THF	50	240	98	95:5
4	*t*-BuOK (1.8)	THF	50	60	98	94:6
5	*t*-BuOK (1.3)	THF	50	60	94 (68)	95:5
6	*n*-BuLi (2.0)	THF	rt	30	93	92:8
7	LiHMDS (2.0)	THF	rt	60	59	92:8
8	K_2_CO_3_ (3.0)	MeCN	60	960	81	91:9
9	Cs_2_CO_3_ (1.8)	MeCN	rt	360	97	93:7
10	KOH (1.3)	THF	rt	7	89	95:5
11	KOH (1.9)	THF	rt	7	92	95:5
12	KOH (1.8)	MeCN	rt	60	90 (67)	97:3
13	KOH (1.8)	MeCN^d^	rt	30	98 (84)	98:2

^a^Determined by GCMS analysis (in brackets isolated yield). ^b^Determined by GCMS analysis of the crude reaction mixture. ^c^Phosphonate **3** was prepared in situ from **1** and diethyl chloromethylphosphonate (−60 °C, 10 min). ^d^Water (8 equiv) was added.

Using optimized reaction conditions (Table 1, entry 13), the scope of the HWE reaction of various aldehydes with phosphonate **3** was explored (Table 2). Aromatic aldehydes with electron-donating groups required longer reaction times than those with electron-acceptor groups. All tested aromatic aldehydes provided compounds **5** in high isolated yields and selectivities. Application of (*E*)-cinnamaldehyde (**3f**) led to the formation of **5f** in only 43% yield. Compound **5f** was configurationally stable in solid form; however, we observed slow isomerization in solution (CDCl_3_) at ambient temperature and in daylight (from *E*/*Z* 93:7 to 66:33 after 10 days). Reactions with ketones, even electrophilic and non-enolizable ones such as 4,4'-dichlorobenzophenone or 2,2,2-trifluoroacetophenone, did not provide the expected alkene products.

**Table 2:** HWE reactions of phosphonate **3** with aldehydes.

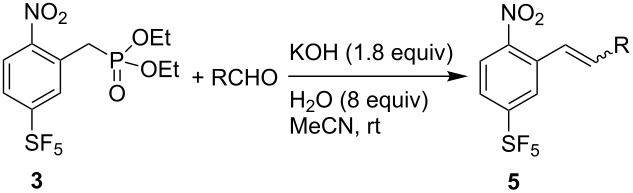				

Entry	R (equiv)	*t* (min)	**5**, Yield (%)^a^	**5**, *E*/*Z*^b^

1	Ph (1.5)	30	**5a**, 84	98:2
2	4-NO_2_C_6_H_4_ (1.7)	5	**5b**, 84	>99:1
3	4-ClC_6_H_4_ (1.1)	30	**5c**, 85	98:2
4	4-MeOC_6_H_4_ (1.5)	90	**5d**, 86	99:1
5	1-Naphthyl (1.2)	30	**5e**, 80	92:8
6	(*E*)-PhCH=CH (1.2)	40	**5f**, 43^c^	93:7^d^
7	Et (1.5)	90	**5g**, 67	94:6

^a^Isolated yield refers to the pure *E*-isomer unless noted otherwise. ^b^Determined by GCMS analysis of the crude reaction mixture. ^c^Isolated yield referring to the 93:7 *E*/*Z* mixture. ^d^This ratio changed to 66:33 upon storage in CDCl_3_ solution at rt for 10 d.

**Scheme 2:**
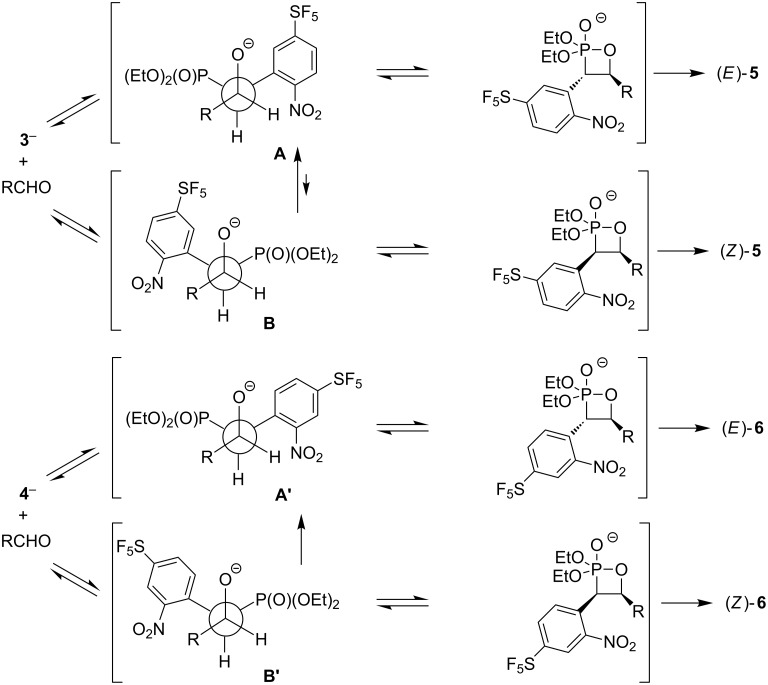
Reactive intermediates involved in HWE reactions to alkenes **5** and **6**.

Next, we investigated analogous HWE reactions of isomeric phosphonate **4** with various aldehydes. Good yields of products **6** were obtained with both aromatic and aliphatic aldehydes. In contrast to most of the reactions with **3**, phosphonate **4** gave exclusively *E*-isomers of **6** (Table 3). This improved selectivity can be explained by relative differences in the stabilities and reactivities of carbanions derived from phosphonates and reactive intermediates. In reactions of phosphoryl-stabilized carbanions with aldehydes, several intermediates are formed reversibly. Less-hindered intermediates **A** and **A’**, which eliminate to *E*-alkene, exist in equilibrium with more-hindered intermediates **B** and **B’** giving *Z*-alkene (Scheme 2). In our reactions, the stabilization of the negative charge in the deprotonated phosphonate is higher for **4** than for **3** due to conjugation of the negative charge with the SF_5_ group in the former case (σ_I_(SF_5_) = 0.55, σ_R_(SF_5_) = 0.11 [5]). Consequently, **4**^−^ is more stable and less nucleophilic than **3**^−^, and therefore, in comparison to the **A**-to-**B** equilibrium the **A’**-to-**B’** equilibrium is shifted more towards **A’** providing only (*E*)*-***6** product (Scheme 2).

**Table 3:** HWE reactions of phosphonate **4** with aldehydes.

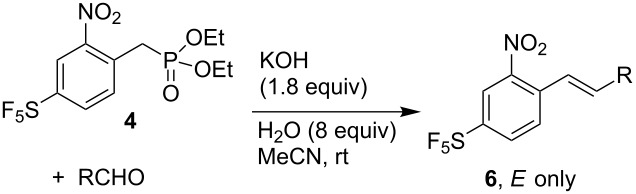			

Entry	R (equiv)	*t* (min)	**6**, Yield (%)^a^

1	4-NO_2_C_6_H_4_ (1.2)	30	**6b**, 97
2	4-ClC_6_H_4_ (1.1)	80	**6c**, 85
3	4-MeOC_6_H_4_ (1.1)	260	**6d**, 76
4	*n*-C_6_H_13_ (1.1)	90	**6h**, 84

^a^Isolated yield.

To demonstrate the versatility of this methodology leading to new carbon-substituted SF_5_-benzenes, several transformations of primary products **5** and **6** were performed. Reduction with hydrogen (1 atm) in the presence of catalytic Raney nickel did not provide full conversion to the respective anilines. Furthermore, the reaction mixtures contained products **7** and **8**. At higher pressure (20 atm), complete nitro group and C=C reduction of stilbenes **5** and **6** to compounds **7** and **8**, respectively, took place (Table 4). Several other conditions were tested, but no system for selective reduction of the nitro group was found.

**Table 4:** Hydrogenation of stilbene derivatives **5** and **6**.

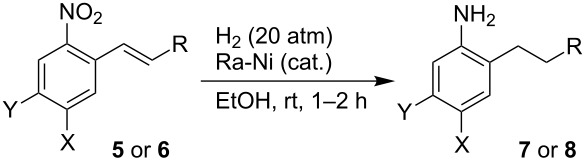					

Entry	**5** or **6**	X	Y	R	**7** or **8**, Yield (%)^a^

1	**5a**	SF_5_	H	Ph	**7a**, 87
2	**6c**	H	SF_5_	4-ClC_6_H_4_	**8c**, 64
3	**6d**	H	SF_5_	4-MeOC_6_H_4_	**8d**, 82

^a^Isolated yield.

**Scheme 3:**
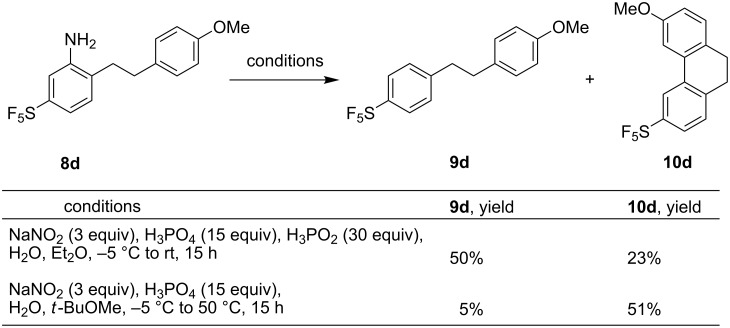
Diazotization/reduction of **8d** to **9d** and the formation of unexpected cyclized product **10d**.

**Scheme 4:**
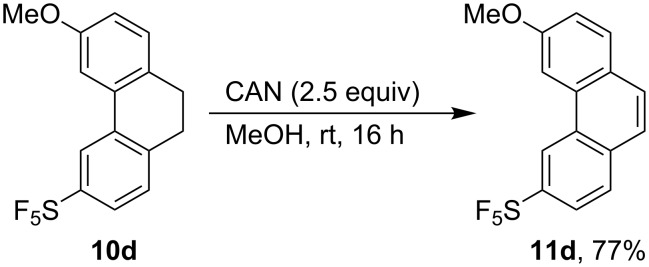
Synthesis of substituted phenanthrene **11d**.

Diazotization was carried out with the aim to prepare 1,2-diarylethane **9d** from aniline **8d** (Scheme 3). A combination of sodium nitrite and phosphoric acid was used. (With HCl, substitution of the amino function by a chlorine atom was observed, and the use of H_2_SO_4_ resulted in low solubility of the formed anilinium in water.) An ether cosolvent (Et_2_O or *t*-BuOMe) improved the yield compared to aqueous or aqueous/THF mixtures. The presence of reducing hypophosphorous acid provided a mixture of the expected **9d** and the cyclized product **10d** resulting from electrophilic aromatic substitution of the substituted phenyl cation intermediate (formed by the decomposition of the diazonium salt), to the electron-rich anisole ring in an unusual *meta*-position relative to the methoxy group. The more activated *para*-position is unavailable, and the reaction in *ortho*-positions would give too strained a product. The formation of the cyclized side product is not restricted to compounds with an electron-donor substituent on the aromatic ring. Similarly to **8d**, the cyclic product was detected by GCMS in diazotization of compound **8c** (product not isolated). Compound **10d** was fully characterized by spectroscopic methods, and the yield was increased to 51% by performing the diazotization reaction in the absence of a reducing reagent (Scheme 3). Aromatization of **10d** by oxidation using CAN was performed to give SF_5_-substituted phenanthrene **11d** in good yield (Scheme 4).

**Scheme 5:**
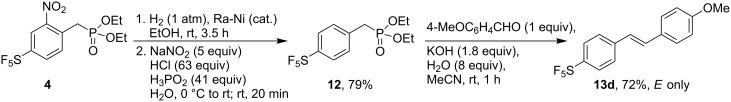
Synthesis of phosphonate **12** and SF_5_-stilbene derivative **13d**.

To avoid problems with alkene reduction and electrophilic aromatic substitution during nitro group removal, we decided to try a different approach to the general synthesis of SF_5_-containing stilbene derivatives, as demonstrated in the synthesis of **13d** shown in Scheme 5. The nitro group in phosphonate **4** was removed by a reduction/diazotization/reduction sequence to give phosphonate **12** in good yield. The following HWE reaction with 4-methoxybenzaldehyde afforded **13d** in good yield, exclusively as the *E*-isomer.

## Conclusion

In conclusion, we have shown access to (*E*)-2-nitro-1-alkenyl-(pentafluorosulfanyl)benzenes from nitro-(pentafluorosulfonyl)benzenes by VNS reaction with diethyl chloromethylphosphonate followed by stereoselective HWE reaction with aldehydes. Reduction of (*E*)-2-nitro-1-alkenyl-(pentafluorosulfanyl)benzenes provided 2-(2-arylethyl)-(pentafluorosulfanyl)anilines, and the formation of (*E*)-1-alkenyl-4-(pentafluorosulfanyl)benzenes was demonstrated from diethyl 4-(pentafluorosulfanyl)benzylphosphonates.

## Supporting Information

File 1 Experimental details and characterization data for all new compounds.

## References

[1] Case J R, Ray N H, Roberts H L (1961). J Chem Soc.

[2] Ray N H (1963). J Chem Soc.

[3] Merrill C I, Cady G H (1961). J Am Chem Soc.

[4] Dudley F B, Cady G H, Eggers D F (1956). J Am Chem Soc.

[5] Kirsch P (2004). Synthesis of complex Organofluorine Compounds. Modern Fluoroorganic Chemistry.

[6] Winter R W, Dodean R A, Gard G L, Soloshonok V A (2005). SF5-Synthons: Pathways to Organic Derivatives of SF6. Fluorine-Containing Synthons.

[7] Kirsch P, Röschenthaler G-V, Soloshonok V A, Mikami K, Yamazaki T (2007). Functional Compounds Based on Hypervalent Sulfur Fluorides. Current Fluoroorganic Chemistry.

[8] Aït-Mohand S, Dolbier W R (2002). Org Lett.

[9] Dolbier W R, Aït-Mohand S, Schertz T D, Sergeeva T A, Cradlebaugh J A, Mitani A, Gard G L, Winter R W, Thrasher J S (2006). J Fluorine Chem.

[10] Bowden R D, Greenhall M P, Moilliet J S, Thomson J (1997). The Preparation Of Fluorinated Organic Compounds. WO Patent.

[11] Bowden R D, Comina P J, Greenhall M P, Kariuki B M, Loveday A, Philp D (2000). Tetrahedron.

[12] Chambers R D, Spink R C H (1999). Chem Commun.

[13] Umemoto T, Garrick L M, Saito N (2012). Beilstein J Org Chem.

[14] Umemoto T, Chika J (2011). Processes for preparing 1,3-dinitro-5-(pentafluorosulfanyl)benzene and its intermediates. U.S. Patent Appl..

[15] Beier P, Pastýříková T, Vida N, Iakobson G (2011). Org Lett.

[16] Beier P, Pastýříková T, Iakobson G (2011). J Org Chem.

[17] Beier P, Pastýříková T (2011). Tetrahedron Lett.

[18] Pastýříková T, Iakobson G, Vida N, Pohl R, Beier P (2012). Eur J Org Chem.

[19] Vida N, Beier P J Fluorine Chem.

[20] Crowley P J, Mitchell G, Salmon R, Worthington P A (2004). Chimia.

[21] Kirsch P, Bremer M, Heckmeier M, Tarumi K (1999). Angew Chem, Int Ed.

[22] Sheppard W A (1962). J Am Chem Soc.

[23] Maryanoff B E, Reitz A B (1989). Chem Rev.

[24] Boutagy J, Thomas R (1974). Chem Rev.

[25] Rein T, Pedersen T M (2002). Synthesis.

[26] Ando K (2000). J Synth Org Chem, Jpn.

[27] Ando K (1997). J Org Chem.

[28] Still W C, Gennari C (1983). Tetrahedron Lett.

