# Supplementary material for: Highly selective synthesis of (E)-alkenyl-(pentafluorosulfanyl)benzenes through Horner–Wadsworth–Emmons reaction
Source: Beilstein J Org Chem. 2012 Jul 25;8:1185–90. doi: 10.3762/bjoc.8.131 (PMC3458736; doi:10.3762/bjoc.8.131)
Supplement: File 1 — Experimental details and characterization data for all new compounds. [file Beilstein_J_Org_Chem-08-1185-s001.pdf]

Supporting Information

for

Highly selective synthesis of (*E*)-alkenyl-(pentafluorosulfanyl)benzenes through Horner–Wadsworth–Emmons reaction

George Iakobson and Petr Beier*

Address: Institute of Organic Chemistry and Biochemistry, Academy of Sciences of the Czech Republic, Flemingovo nám. 2, 166 10 Prague, Czech Republic

Email: Petr Beier - beier@uochb.cas.cz

Experimental details and characterization data for all new compounds

1. General experimental procedures

Infrared spectra were measured on an FTIR instrument. NMR spectra were recorded at 300 K on Bruker Avance 400, 500 or 600 MHz instruments. Chemical shifts (δ) are reported in ppm relative to Me_4Si (0 ppm, for ^{1}H NMR), residual $CHCl_3$ (7.26 ppm for ^{1}H NMR), $CDCl_3$ (77.0 ppm for ^{13}C NMR), residual $HD_2CC(O)CD_3$ (2.05 ppm for ^{1}H NMR), $CD_3C(O)CD_3$ (29.8 ppm for ^{13}C NMR), residual $HD_2CS(O)CD_3$ (2.50 ppm for ^{1}H NMR), $CD_3S(O)CD_3$ (39.4 ppm for ^{13}C NMR), internal $CFCl_3$ (0 ppm for ^{19}F NMR), and external H_3PO_4 in water (0 ppm for ^{31}P NMR). GCMS spectra were recorded on an Agilent 7890A gas chromatograph coupled using a 5% phenyl methyl siloxane (30 m x 250 μm with a film thickness of 0.25 μm) GC column with a 5975C quadrupole mass-selective electron impact (EI) detector (70 eV). High-resolution mass spectra (HRMS) were recorded on an Agilent 7890A gas chromatograph coupled with a Waters GCT Premier orthogonal acceleration time-of-flight detector using electron impact (EI) or chemical (CI) ionizations or on a LTQ Orbitrap XL instrument using electrospray (ESI) ionization. THF was dried by distillation from Na/benzophenone, DMF and MeCN were dried using molecular sieves (3 Å). For the HWE reactions, regular (not dried) MeCN containing 0.1% of water was used.

2. General procedure for the synthesis of compounds 5 and 6

A solution of phosphonate **3** or **4** (100 mg, 0.25 mmol) and aldehyde (0.25–0.38 mmol, 1.0–1.5 equiv) in MeCN (0.25 mL) was added to a mixture of KOH (25 mg, 0.45 mmol, 1.8 equiv) in MeCN (2.5 mL) and water (36 µL, 8 equiv). The resulting mixture was stirred at rt for a given time (until disappearance of the violet color), followed by the addition of water (6 mL) and removal of MeCN under reduced pressure. The product was purified by one of these methods: Method A – the precipitate was filtered off, washed with water and dried under reduced pressure; Method B – the crude product was extracted into Et_2O (3 × 20 mL), the combined organic phase was washed with water (20 mL) and brine (20 mL), and dried ($MgSO_4$), and the solvent was removed under reduced pressure. The pure product was obtained by recrystallization; Method C – the crude product was extracted into Et_2O (3 × 20 mL), the combined organic phase was washed with water (20 mL) and brine (20 mL), and dried ($MgSO_4$), and the solvent was removed under reduced pressure. The pure product was obtained by column chromatography using silica gel 60.

2.1. (*E*)-1-Nitro-2-styryl-4-(pentafluorosulfanyl)benzene (5a). Prepared according to the general procedure from **3** (100 mg, 0.25 mmol) and benzaldehyde (40 µL, 0.38 mmol, 1.5 equiv) in 30 min giving **5a** as a yellow solid (74 mg, 84%) using purification Method B; mp 129.8–132.5 °C (hexane); R_f 0.38 (hexane–EtOAc, 95:5); IR (KBr) ν_{max} (cm^{-1}) 3118, 3089, 3064, 3030, 1630, 1608, 1578, 1523, 1347, 852; ^1H NMR (400 MHz, $CDCl_3$) δ 7.14 (d, 1H, $^3J_{HH}$ = 16.2 Hz), 7.32–7.44 (m, 3H), 7.50 (d, 1H, $^3J_{HH}$ = 16.2 Hz), 7.54 (m, 2H), 7.76 (dd, 1H, $^3J_{HH}$ = 8.9 Hz, $^4J_{HH}$ = 2.4 Hz), 7.99–8.04 (m, 1H), 8.13 (d, 1H, $^4J_{HH}$ = 2.3 Hz); ^{13}C NMR (151 MHz, $CDCl_3$) δ 121.4, 125.2, 125.3 (quin, $^3J_{CF}$ = 4.5 Hz), 125.9 (quin, $^3J_{CF}$ = 4.5 Hz), 127.3, 128.9, 129.3, 133.8, 135.6, 136.1, 148.8, 156.3 (quin, $^2J_{CF}$ = 18.8 Hz); ^{19}F NMR (377 MHz, $CDCl_3$) δ 62.0 (d, 4F, $^2J_{FF}$ = 150.3 Hz), 80.2–81.9 (m, 1F); MS (EI) *m*/*z*: (rel. int.) 351 (16) $[M]^+$, 334 (41), 245 (100), 176 (40), 105 (42), 91 (71); HRMS (EI) *m*/*z* calcd for $C_{14}H_{10}F_5NO_2S$ $[M]^+$ 351.0350; found, 351.0352.

2.2. (*E*)-1-Nitro-2-(4-nitrostyryl)-4-(pentafluorosulfanyl)benzene (5b). Prepared according to the general procedure from **3** (100 mg, 0.25 mmol) and 4-nitrobenzaldehyde (42 mg, 0.28 mmol, 1.1 equiv) in 5 min giving **5b** as a pale yellow solid (83 mg, 84%) using purification Method A; mp 226–230 °C; R_f 0.35 (hexane–acetone, 80:20); IR (KBr) ν_{max} (cm^{-1}) 3097, 1605, 1595, 1534, 1517, 1494, 1314, 831; ^1H NMR (600 MHz, acetone-d_6) δ 7.69 (d, 1H, $^3J_{HH}$ = 16.3 Hz), 7.79 (d, 1H, $^3J_{HH}$ = 16.3 Hz), 7.94–7.98 (m, 2 H), 8.13 (dd, 1H,

$^{3}J_{HH}$ = 8.9 Hz, $^{4}J_{HH}$ = 2.4 Hz), 8.27 (br d, 1H, $^{3}J_{HH}$ = 8.9 Hz), 8.28–8.32 (m, 2H), 8.51 (d, 1H, $^{4}J_{HH}$ = 2.3 Hz); ^{13}C NMR (151 MHz, acetone-d_6) δ 124.0, 125.8, 125.8, 126.2 (quin, $^{3}J_{CF}$ = 4.5 Hz), 126.8 (quin, $^{3}J_{CF}$ = 4.5 Hz), 128.2, 132.9, 133.5, 142.7, 147.8, 117.9, 155.6–156.3 (m), 205.2; ^{19}F NMR (377 MHz, acetone-d_6) δ 63.2 (d, 4F, $^{2}J_{FF}$ = 149.2 Hz), 81.5–83.4 (m, 1F); MS (EI) *m*/*z* (rel. int.) 396 (5) $[M]^+$, 379 (15), 245 (100), 218 (36); HRMS (EI) *m*/*z* calcd for $C_{14}H_9F_5O_4S$ $[M]^+$ 396.0203; found, 396.0197.

2.3. (*E*)-1-Nitro-2-(4-chlorostyryl)-4-(pentafluorosulfanyl)benzene (5c). Prepared according to the general procedure from **3** (100 mg, 0.25 mmol) and 4-chlorobenzaldehyde (39 mg, 0.28 mmol, 1.1 equiv) in 30 min giving **5c** as a yellow solid (82 mg, 85%) using purification Method B; mp 157.5–160.3 °C (hexane); R_f 0.53 (hexane–acetone, 80:20); IR (KBr) ν_{max} (cm^{-1}) 1628, 1606, 1591, 1574, 1563, 1530, 1491, 835, 811; ^{1}H NMR (500 MHz, DMSO-d_6) δ 7.42 (d, 1H, $^{3}J_{HH}$ = 16.3 Hz), 7.45–7.49 (m, 2H), 7.54 (d, 1H, $^{3}J_{HH}$ = 16.3 Hz), 7.65–7.69 (m, 2H), 8.06 (dd, 1H, $^{3}J_{HH}$ = 9.0 Hz, $^{4}J_{HH}$ = 2.4 Hz), 8.17–8.20 (m, 1H), 8.43 (d, 1H, $^{4}J_{HH}$ = 2.4 Hz); ^{13}C NMR (126 MHz, DMSO-d_6) δ 121.8, 125.5 (quin, $^{3}J_{CF}$ = 4.5 Hz), 125.6–126.1 (m), 128.9, 129.0, 132.8, 133.5, 134.3, 134.8, 149.2, 154.7–155.3 (m); ^{19}F NMR (470 MHz, DMSO-d_6) δ 64.2 (d, 4F, $^{2}J_{FF}$ = 151.6 Hz), 84.0–85.4 (m, 1F); MS (EI) *m*/*z* (rel. int.) 385 (21) $[M]^+$, 370 (15), 368 (40), 245 (100), 218 (49), 176 (49), 139 (38), 125 (54), 110 (33); HRMS (EI) *m*/*z* calcd for $C_{14}H_9ClF_5NO_2S$ $[M]^+$ 384.9961; found, 384.9963.

2.4. (*E*)-1-Nitro-2-(4-methoxystyryl)-4-(pentafluorosulfanyl)benzene (5d). Prepared according to the general procedure from **3** (100 mg, 0.25 mmol) and 4-methoxybenzaldehyde (46 µL, 0.38 mmol, 1.5 equiv) in 90 min giving **5d** as an orange solid (79 mg, 86%) using purification Method C; mp 126–132 °C (EtOH); R_f 0.25 (hexane–EtOAc, 95:5); IR (KBr) ν_{max} (cm^{-1}) 2839, 1629, 1602, 1576, 1528, 1513, 1352, 1255, 962, 851; ^{1}H NMR (500 MHz, $CDCl_3$) δ 3.84 (s, 3H), 6.91–6.94 (m, 2H), 7.11 (d, 1H, $^{3}J_{HH}$ = 16.1 Hz), 7.38 (d, 1H, $^{3}J_{HH}$ = 16.1 Hz), 7.47–7.52 (m, 2H), 7.72 (dd, 1H, $^{3}J_{HH}$ = 8.9 Hz, $^{4}J_{HH}$ = 2.4 Hz), 7.70–7.74 (m, 1H), 8.12 (d, 1H, $^{4}J_{HH}$ = 2.4 Hz); ^{13}C NMR (126 MHz, $CDCl_3$) δ 55.4, 114.4, 119.0, 124.8 (quin, $^{3}J_{CF}$ = 4.5 Hz), 125.2, 125.6 (quin, $^{3}J_{CF}$ = 4.5 Hz), 128.4, 128.8, 134.1, 135.8, 148.6, 155.9–156.6 (m), 160.6; ^{19}F NMR (470 MHz, $CDCl_3$) δ 62.0 (d, 4F, $^{2}J_{FF}$ = 150.6 Hz), 80.6–81.9 (m, 1F); MS (EI) *m*/*z* (rel. int.) 381 (28) $[M]^+$, 364 (16), 136 (58), 135 (100), 121 (36); HRMS (EI) *m*/*z* calcd for $C_{15}H_{12}F_5NO_3S$ $[M]^+$ 381.0453; found, 381.0458.

2.5. (*E*)-1-(2-Nitro-5-(pentafluorosulfanyl)styryl)naphthalene (5e). Prepared according to the general procedure from **3** (100 mg, 0.25 mmol) and 1-naphthylaldehyde (41 µL, 0.30 mmol, 1.2 equiv) in 30 min giving **5e** as a yellow solid (80 mg, 80%) using purification Method B; mp 170–174 °C (EtOH); R_f 0.34 (hexane–acetone, 97.5:2.5); IR (KBr) ν_{max} (cm^{-1}) 3082, 1605, 1570, 1539, 1509, 1353, 839, 824; ^1H NMR (500 MHz, $CDCl_3$) δ 7.50–7.61 (m, 4H), 7.77 (dt, 1H, $^3J_{HH}$ = 7.2 Hz, $^5J_{HH}$ = 0.9 Hz), 7.81 (dd, 1H, $^3J_{HH}$ = 9.0 Hz, $^4J_{HH}$ = 2.3 Hz), 7.87–7.92 (m, 3H), 8.04 (br d, 1H, $^3J_{HH}$ = 8.9 Hz), 8.15–8.18 (m, 1H), 8.23 (d, 1H, $^4J_{HH}$ = 2.4 Hz); ^{13}C NMR (126 MHz, $CDCl_3$) δ 123.3, 124.6, 124.8, 125.3, 125.5–125.7 (m), 126.2, 126.4 (quin, $^3J_{CF}$ = 4.5 Hz), 126.7, 128.8, 129.7, 131.2, 133.2, 133.3, 133.7, 134.1, 148.8, 154.6–155.2 (m); ^{19}F NMR (470 MHz, $CDCl_3$) δ 62.1 (d, 4F, $^2J_{FF}$ = 150.7 Hz), 80.4–81.7 (m, 1F); MS (EI) *m*/*z* (rel. int.) 401 (68) $[M]^+$, 372 (23), 226 (51), 156 (100), 155 (68), 141 (43), 128 (74); HRMS (EI) *m*/*z* calcd for $C_{18}H_{12}$ F_5NO_2S $[M]^+$ 401.0509; found, 401.0507.

2.6. 1-Nitro-2-(4-phenylbuta-1,2-dien-1-yl)-4-(pentafluorosulfanyl)benzene (5f). Prepared according to the general procedure from **3** (100 mg, 0.25 mmol) and (*E*)-cinnamaldehyde (38 µL, 0.30 mmol, 1.2 equiv) in 40 min giving **5f** as a pale orange solid (41 mg, 43%, *E*:*Z* 93:3 reducing to 66:33 upon storage in $CDCl_3$ solution at rt for 10 d) using purification Method C; R_f 0.40 (hexane–acetone, 97.5:2.5); IR (KBr) ν_{max} (cm^{-1}) 3110, 3084, 3061, 3028, 1624, 1605, 1530, 1494, 1348, 849, 813; **major** (1*E*,3*E*)-**5f:** ^1H NMR (400 MHz, $CDCl_3$) δ 6.83–6.90 (m, 1H), 6.94–7.13 (m, 3H), 7.26–7.41 (m, 3H), 7.44–7.51 (m, 2H), 7.71 (dd, 1H, $^3J_{HH}$ = 8.9 Hz, $^4J_{HH}$ = 2.3 Hz), 7.93–7.97 (m, 1H), 8.09 (d, 1H, $^4J_{HH}$ = 2.3 Hz); ^{13}C NMR (126 MHz, $CDCl_3$) δ 124.3, 125.0 (quin, $^3J_{CF}$ = 4.4 Hz), 125.2, 125.4 (quin, $^3J_{CF}$ = 4.5 Hz), 126.9, 127.8, 128.7, 128.8, 133.6, 136.4, 136.5, 137.5, 148.5, 155.9–156.5 (m); ^{19}F NMR (377 MHz, $CDCl_3$) δ 61.9 (d, 4F, $^2J_{FF}$ = 150.3 Hz), 80.3–82.0 (m, 1F); MS (EI) *m*/*z* (rel. int.) 377 (22) $[M]^+$, 376 (19), 348 (49), 202 (57), 131 (100), 117 (38), 115 (92), 105 (63), 91 (48); HRMS (CI) *m*/*z* calcd for $C_{16}H_{13}$ F_5O_2S $[MH]^+$ 378.0587; found, 378.0591; **minor** (1*Z*,3*E*)-**5f:** ^1H NMR (400 MHz, $CDCl_3$) δ 6.64–6.70 (m, 1H), 6.75–6.79 (m, 1H), 6.81–6.86 (m, 1H), 6.92–6.98 (m, 1H), 7.26–7.31 (m, 2H), 7.32–7.37 (m, 2H), 7.83 (dd, 1H, $^3J_{HH}$ = 9.0 Hz, $^4J_{HH}$ = 2.4 Hz), 7.98 (d, 1H, $^4J_{HH}$ = 2.3 Hz), 8.11–8.14 (m, 1H); ^{13}C NMR (126 MHz, $CDCl_3$) δ 122.5, 123.2, 125.3, 125.6 (quin, $^3J_{CF}$ = 4.4 Hz), 126.8, 128.6, 128.8, 130.4 (quin, $^3J_{CF}$ = 4.5 Hz), 133.5, 134.4, 136.3, 138.4, 149.0, $C_{Ar}SF_5$ not detected; ^{19}F NMR (377 MHz, $CDCl_3$) δ 62.1 (d, 4F, $^2J_{FF}$ = 150.3 Hz), 80.1–81.9 (m, 1 F).

2.7. 2-(But-1-en-1yl)-1-nitro-4-(pentafluorosulfanyl)benzene (5g). Prepared according to the general procedure from **3** (100 mg, 0.25 mmol) and propionaldehyde (27 µL, 0.38 mmol, 1.5 equiv) in 90 min giving **5g** as a yellow solid (51 mg, 67%, *E*:*Z* 94:6) using purification Method C; mp 38–42 °C (hexane); R_f 0.16 (hexane); IR (KBr) ν_{max} (cm^{-1}) 3113, 3095, 3060, 2972, 2938, 2879, 1646,1609, 1575, 1352, 1354, 850, 818; ^{1}H NMR (500 MHz, $CDCl_3$) δ 1.14 (t, 3H, $^3J_{HH}$ = 7.4 Hz), 2.29–2.36 (m, 2H), 6.39 (dt, 1H, $^3J_{HH}$ = 15.7 Hz, $^4J_{HH}$ = 6.4 Hz), 6.80 (dt, 1H, $^3J_{HH}$ = 15.7 Hz, $^4J_{HH}$ = 1.7 Hz), 7.72 (dd, 1H, $^3J_{HH}$ = 8.9 Hz, $^4J_{HH}$ = 2.4 Hz), 7.90–7.94 (m, 1H), 7.97 (d, 1H, $^4J_{HH}$ = 2.4 Hz); ^{13}C NMR (126 MHz, $CDCl_3$) δ 122.4, 124.8, 124.9 (quin, $^3J_{CF}$ = 4.5 Hz), 126.2 (quin, $^3J_{CF}$ = 4.8 Hz), 134.2, 141.0, 148.5, 156.1 (quin, $^3J_{CF}$ = 18.8 Hz); ^{19}F NMR (470 MHz, $CDCl_3$) δ 62.0 (d, 4F, $^2J_{FF}$ = 150.7 Hz), 80.6–81.9 (m, 1F); MS (EI) *m*/*z* (rel. int.) 303 (4) $[M]^+$, 246 (74), 131 (53), 130 (39), 128 (38), 115 (44), 89 (41), 57 (100); HRMS (EI) *m*/*z* calcd for $C_{13}H_{10}F_5O_2S_2$ $[M]^+$ 303.0352; found, 303.0353.

2.8. (*E*)-2-Nitro-1-(4-nitrostyryl)-4-(pentafluorosulfanyl)benzene (6b). Prepared according to the general procedure from **4** (100 mg, 0.25 mmol) and 4-nitrobenzaldehyde (45 mg, 0.30 mmol, 1.2 equiv) in 30 min giving **6b** as a pale yellow solid (96 mg, 97%); mp 138–140 °C; R_f 0.29 (hexane–acetone, 80:20); IR (KBr) ν_{max} (cm^{-1}) 3111, 3084, 1607, 1599, 1565, 1520, 1532, 1345, 848; ^{1}H NMR (500 MHz, acetone-d_6) δ 7.62 (d, 1H, $^3J_{HH}$ = 16.3 Hz), 7.83 (d, 1H, $^3J_{HH}$ = 16.2 Hz), 7.94–7.97 (m, 2H), 8.27 (d, 2H, $^4J_{HH}$ = $^5J_{HH}$ = 1.5 Hz), 8.28–8.31 (m, 2H), 8.52 (t, 1H, $^4J_{HH}$ = $^5J_{HH}$ = 1.4 Hz); ^{13}C NMR (126 MHz, acetone-d_6) δ 122.8 (quin, $^3J_{CF}$ = 4.9 Hz), 124.0, 126.0, 128.3, 129.7, 130.3 (quin, $^3J_{CF}$ = 4.5 Hz), 134.1, 135.5, 142.5, 147.8, 151.7–152.4 (m), 205.2; ^{19}F NMR (367 MHz, acetone-d_6) δ 63.5 (d, 4F, $^2J_{FF}$ = 150.3 Hz), 81.7–83.4 (m, 1F); MS (EI) *m*/*z* (rel. int.) 396 (4) $[M]^+$, 379 (15), 245 (100), 218 (37); HRMS (EI) *m*/*z* calcd for $C_{14}H_9F_5N_2O_4S$ $[M]^+$ 396.0203; found, 396.0205.

2.9. (*E*)-2-Nitro-1-(4-chlorostyryl)-4-(pentafluorosulfanyl)benzene (6c). Prepared according to the general procedure from **4** (100 mg, 0.25 mmol) and 4-chlorobenzaldehyde (39 mg, 0.28 mmol, 1.1 equiv) in 80 min giving **6c** as a yellow solid (82 mg, 85%) using purification Method C; mp 112.0–113.0 °C (EtOH); R_f 0.39 (hexane–acetone, 95:5); IR (KBr) ν_{max} (cm^{-1}) 3112, 1629, 1608, 1592, 1531, 1493, 1349, 849, 821; ^{1}H NMR (500 MHz, $CDCl_3$) δ 7.12 (d, 1H, $^3J_{HH}$ = 16.1 Hz), 7.34–7.40 (m, 2H), 7.45–7.51 (m, 2H), 7.57 (d, 1H, $^3J_{HH}$ = 16.1 Hz), 7.84–7.88 (m, 1H), 7.96 (dd, 1H, $^3J_{HH}$ = 8.7 Hz, $^4J_{HH}$ = 2.1 Hz), 8.38 (d, 1H, $^4J_{HH}$ = 2.3 Hz); ^{13}C NMR (126 MHz, $CDCl_3$) δ 122.2, 123.2 (quin, $^3J_{CF}$ = 4.8 Hz), 128.5, 128.6, 129.2, 130.1 (quin, $^3J_{CF}$ = 4.5 Hz), 134.2, 135.3, 135.5, 136.0, 146.9, 151.1 (quin, $^2J_{CF}$ =

20.2 Hz); ^{19}F NMR (377 MHz, $CDCl_3$) δ 62.6 (d, 4F, $^2J_{FF}$ = 151.4 Hz), 80.2–81.9 (m, 1F); MS (EI) *m/z* (rel. int.) 385 (14) $[M]^+$, 370 (12), 368 (31), 245 (100), 218 (49), 176 (48), 139 (42), 138 (44), 125 (53); HRMS (EI) *m/z* calcd for $C_{14}H_9ClF_5NO_2S$ $[M]^+$ 384.9963; found, 384.9969.

2.10. (*E*)-2-Nitro-1-(4-methoxystyryl)-4-(pentafluorosulfanyl)benzene (6d). Prepared according to the general procedure from **4** (100 mg, 0.25 mmol) and 4-methoxybenzaldehyde (33 µL, 0.28 mmol, 1.1 equiv) in 260 min giving **6d** as yellow needles (70 mg, 76%) using purification Method C; mp 117.8–120.0 °C; R_f 0.16 (hexane–EtOAc, 95:5); IR (KBr) ν_{max} (cm^{-1}) 3112, 3035, 3009, 2841, 1624, 1600, 1575, 1531, 1513, 1349, 1256, 906, 848, 831; ^{1}H NMR (600 MHz, $CDCl_3$) δ 3.84 (s, 3H), 6.90–6.93 (m, 2H), 7.15 (d, 1H, $^3J_{HH}$ = 16.1 Hz), 7.45 (dt, 1H, $^3J_{HH}$ = 16.1 Hz, $^5J_{HH}$ = 0.6 Hz), 7.47 (m, 2H), 7.83–7.86 (m, 1H), 7.91 (ddd, 1H, $^3J_{HH}$ = 8.8 Hz, $^4J_{HH}$ = 2.3 Hz, $^5J_{HH}$ = 0.6 Hz), 8.33 (dd, 1H, $^4J_{HH}$ = 2.3 Hz, $^5J_{HH}$ = 0.3 Hz); ^{13}C NMR (151 MHz, $CDCl_3$) δ 55.3, 114.4, 119.0, 123.1 (quin, $^3J_{CF}$ = 4.8 Hz), 128.0, 128.5, 128.9, 129.8 (quin, $^3J_{CF}$ = 4.3 Hz), 136.5, 136.6, 146.6, 151.4 (quin, $^2J_{CF}$ = 19.9 Hz), 160.8; ^{19}F NMR (377 MHz, $CDCl_3$) δ 62.6 (m, 4F, $^2J_{FF}$ = 151.4 Hz), 80.6–82.4 (m, 1F); MS (EI) *m/z* (rel. int.) 381 (20) $[M]^+$, 364 (15), 136 (59), 135 (100); HRMS (EI) *m/z* calcd for $C_{15}H_{12}F_5NO_3S$ $[M]^+$ 381.0458; found, 381.0466.

2.11. (*E*)-2-Nitro-1-(oct-1-en-1-yl)-4-(pentafluorosulfanyl)benzene (6h). Prepared according to the general procedure from **4** (100 mg, 0.25 mmol) and heptanal (54 µL, 0.38 mmol, 1.5 equiv) in 90 min giving **6h** as a pale yellow liquid (76 mg, 84%) using purification Method C; R_f 0.25 (hexane); IR (KBr) ν_{max} (cm^{-1}) 3115, 2959, 2931, 2858, 1610, 1566, 1531, 1351, 906, 852, 838; ^{1}H NMR (600 MHz, $CDCl_3$) δ 0.90 (t, 1H, $^3J_{HH}$ = 7.0 Hz), 1.28–1.40 (m, 6H), 1.49–1.55 (m, 2H), 2.29–2.34 (m, 2H), 6.39 (dt, 1H, $^2J_{HH}$ = 15.6 Hz, $^3J_{HH}$ = 7.0 Hz), 6.87 (dm, 1H, $^3J_{HH}$ = 15.6 Hz), 7.70–7.73 (m, 1H), 7.88 (ddd, 1H, $^3J_{HH}$ = 8.7 Hz, $^4J_{HH}$ = 2.3 Hz, $^5J_{HH}$ = 0.6 Hz), 8.28 (dd, 1H, $^4J_{HH}$ = 2.3 Hz, $^5J_{HH}$ = 0.3 Hz); ^{13}C NMR ($CDCl_3$) δ 14.0, 22.6, 28.7, 28.8, 31.6, 33.3, 122.7 (quin, $^3J_{CF}$ = 4.8 Hz), 123.5, 128.7, 129.7 (quin, $^3J_{CF}$ = 4.5 Hz), 136.6, 140.6, 146.6, 151.6 (quin, $^2J_{CF}$ = 19.9 Hz); ^{19}F NMR ($CDCl_3$) δ 63.1 (d, 4F, $^2J_{FF}$ = 151.4 Hz), 81.0–82.8 (m, 1F); MS (EI) *m/z* (rel. int.) 340 (14), 273 (33), 272 (41), 246 (60) 115 (46), 113 (50) 55 (56), 43 (100), 41 (46); HRMS (EI) *m/z* calcd for $C_{14}H_{18}F_5NO_2S$ $[M]^+$ 359.0978; found, 359.0990.

3. General procedure for the synthesis of compounds 7 and 8

A suspension of Raney nickel (20–100 mg) in water was washed with ethanol (2 × 10 mL). A solution of **5** or **6** (0.14–0.68 mmol) in EtOH (10 mL) was added and the mixture was hydrogenated at 20 atm of H_2 during 1–2 h, followed by filtration, washing with THF (3 × 10 mL), and concentration of the filtrate under reduced pressure. The resulting residue was purified by column chromatography (silica gel 60 or aluminum oxide Brockmann I alkaline) to give pure compounds **7** or **8**.

3.1. 2-Phenethyl-4-(pentafluorosulfanyl)aniline (7a). Prepared according to the general procedure from **5a** (50 mg, 0.14 mmol) in 2 h giving **7a** as a pale brown liquid (40 mg, 87%) using purification by column chromatography (silica gel, hexane–CH_2Cl_2, 70:30); mp 129.8–132.5 °C; R_f 0.38 (hexane–CH_2Cl_2, 60:40); IR (KBr) ν_{max} (cm^{-1}) 3498, 3406, 3104, 3087, 3064, 3029, 3004, 1627, 1603, 1580, 1501, 1308, 1102, 835; ^{1}H NMR (400 MHz, $CDCl_3$) δ 2.73–2.80 (m, 2H), 2.89–2.95 (m, 2H), 3.72–3.92 (br s, 2H), 6.58 (d, 1H, $^3J_{HH}$ = 8.6 Hz), 7.13–7.19 (m, 2H), 7.19–7.25 (m, 1H), 7.25–7.33 (m, 2H), 7.36 (d, 1H, $^4J_{HH}$ = 2.5 Hz), 7.40 (dd, 1H, $^3J_{HH}$ = 8.6 Hz, $^4J_{HH}$ = 2.5 Hz); ^{13}C NMR (126 MHz, $CDCl_3$) δ 33.3, 34.7, 114.0, 124.7, 125.1 (quin, $^3J_{CF}$ = 4.5 Hz), 126.3, 127.2 (quin, $^3J_{CF}$ = 4.8 Hz), 128.4, 128.6, 140.9, 144.3–145.0 (m), 146.7; ^{19}F NMR (377 MHz, $CDCl_3$) δ 64.2 (d, 4F, $^2J_{FF}$ = 150.3 Hz), 86.4–88.6 (m, 1F); MS (EI) *m*/*z* (rel. int.) 323 (26) $[M]^+$, 232 (100); HRMS (EI) *m*/*z* calcd for $C_{14}H_{14}F_5NS$ $[M]^+$ 323.0767; found, 323.0772.

3.2. 2-(2-(4-Chlorophenyl)ethyl]-5-(pentafluorosulfanyl)aniline (8c). Prepared according to the general procedure from **6c** (262 mg, 0.679 mmol) in 1 h giving **8c** as a white solid (154 mg, 64%) using purification by column chromatography (aluminum oxide, hexane–EtOAc, 85:15); mp 75–80 °C; R_f 0.13 (hexane–CH_2Cl_2, 70:30); IR (KBr) ν_{max} (cm^{-1}) 3465, 3384, 3084, 3066, 3048, 3027, 1629, 1602, 1581, 1508, 1491, 841, 806; ^{1}H NMR (400 MHz, $CDCl_3$) δ 2.73–2.78 (m, 2H), 2.86–2.92 (m, 2H), 3.68 (br s, 2H), 6.99–7.11 (m, 5H), 7.23–7.27 (m, 2H); ^{13}C NMR (126 MHz, $CDCl_3$) δ 32.7, 33.8, 112.6 (quin, $^3J_{CF}$ = 4.8 Hz), 115.9 (quin, $^3J_{CF}$ = 4.8 Hz), 128.6, 128.8, 129.3, 129.7, 132.0, 139.4, 144.2, 152.9 (quin, $^2J_{CF}$ = 16.6 Hz); ^{19}F NMR (470 MHz, $CDCl_3$) δ 62.5 (d, 4F, $^2J_{FF}$ = 149.8 Hz), 84.5–86.0 (m, 1F); MS (EI) *m*/*z* (rel. int.) 359 (6), 357 (16) $[M]^+$, 232 (100); HRMS (EI) *m*/*z* calcd for $C_{14}H_{13}ClF_5NS$ $[M]^+$ 357.0377; found, 357.0371.

3.3. 2-(2-(4-Methoxyphenyl)ethyl]-5-(pentafluorosulfanyl)aniline (8d). Prepared according to the general procedure from **6d** (160 mg, 0.420 mmol) in 2 h giving **8d** as a white solid (121 mg, 82%) using purification by column chromatography (aluminum oxide, hexane–EtOAc, 85:15); mp 83.5–85.7 °C; R_f 0.29 (hexane–CH_2Cl_2, 60:40); IR (KBr) ν_{max} (cm^{-1}) 3510, 3475, 3385, 3102, 3069, 3035, 3013, 2959,2940, 2865, 2842, 1632, 1611, 1584, 1510, 124, 1031, 851; ^{1}H NMR (500 MHz, $CDCl_3$) δ 2.72–2.77 (m, 2H), 2.83–2.88 (m, 2H), 3.64 (br s, 2H), 3.78 (s, 3H), 6.80–6.87 (m, 2H), 7.01 (d, 1H, $^4J_{HH}$ = 2.1 Hz), 7.03 (d, 1H, $^3J_{HH}$ = 8.4 Hz), 7.06 (d, 1H, $^4J_{HH}$ = 2.1 Hz), 7.06–7.09 (m, 1H); ^{13}C NMR (126 MHz, $CDCl_3$) δ 33.1, 33.8, 55.2, 112.5 (quin, $^3J_{CF}$ = 4.6 Hz), 113.9, 115.8 (quin, $^3J_{CF}$ = 4.6 Hz, 129.3, 129.4, 133.1, 144.4, 152.7 (quin, $^2J_{CF}$ = 16.5 Hz), 158.1; ^{19}F NMR (377 MHz, $CDCl_3$) δ 62.5 (d, $^2J_{FF}$ = 150.3 Hz, 4F), 84.6–86.2 (m, 1F); MS (EI) *m/z* (rel. int.) 353 (9) $[M]^+$, 121 (100); HRMS (EI) *m/z* calcd for $C_{15}H_{16}F_5NOS$ $[M]^+$ 353.0873; found, 323.0880.

4. Synthesis of 1-methoxy-4-(4-(pentafluorosulfanyl)phenethyl)benzene (9d). A solution of $NaNO_2$ (70 mg, 1.0 mmol, 3 equiv) in water (0.4 mL) cooled to 0 °C was added dropwise to a stirred mixture of **8d** (120 mg, 0.34 mmol), Et_2O (2 mL) and aqueous 85% H_3PO_4 (588 mg, 5.1 mmol, 15 equiv) cooled to –5 °C. After the mixture was stirred for a few minutes at this temperature, aqueous 50% H_3PO_2 (1.35 g, 10.2 mmol, 30 equiv) was added, and the mixture was slowly warmed to rt and stirred for 15 h. Water (15 mL) was added and the product was extracted into Et_2O (3 × 10 mL). The combined organic phase was washed with brine (10 mL) and dried ($MgSO_4$), and the solvent was removed under reduced pressure. ^{19}F NMR spectrum indicated **9d**:**10d** ratio of 2.27:1. Purification by column chromatography using silica gel 60 gave **9d** as a colorless liquid (57 mg, 50%); R_f 0.31 (hexane–CH_2Cl_2, 80:20); IR (KBr) ν_{max} (cm^{-1}) 2839, 1629, 1602, 1576, 1528, 1513, 1352, 1255, 1176, 851, 851; ^{1}H NMR (500 MHz, $CDCl_3$) δ 2.84–2.89 (m, 2H), 2.91–2.96 (m, 2H), 3.79 (s, 3H), 6.81–6.84 (m, 2H), 7.04–7.08 (m, 2H), 7.22 (br d, 2H, $^3J_{HH}$ = 8.2 Hz), 7.62–7.66 (m, 2H); ^{13}C NMR (125.7 MHz, $CDCl_3$) δ 36.4, 37.6, 55.2, 113.8, 125.9 (quin, $^3J_{CF}$ = 4.5 Hz), 128.7, 129.3, 132.8, 145.8, 151.9 (quin, $^2J_{CF}$ = 17.3 Hz), 158.0; ^{19}F NMR (470.3 MHz, $CDCl_3$) δ 72.7 (d, $^2J_{FF}$ = 149.7 Hz), 84.1–85.5 (m, 1F); MS (EI) *m/z* (rel. int.) 338 (4) $[M]^+$, 121 (100); HRMS (EI) *m/z* calcd for $C_{15}H_{15}F_5OS$ $[M]^+$ 338.0764; found, 338.0760.

5. Synthesis of 3-methoxy-6-(pentafluorosulfanyl)-9,10-dihydrophenanthrene (10d). A solution of $NaNO_2$ (47 mg, 0.68 mmol, 3 equiv) in water (0.3 mL) cooled to 0 °C was added dropwise to a stirred mixture of **8d** (80 mg, 0.27 mmol), *t*-BuOMe (2 mL) and aqueous 85% H_3PO_4 (400 mg, 6.9 mmol, 15 equiv) cooled to –5 °C. The mixture was slowly warmed to 50 °C and stirred for 15 h. Water (15 mL) was added and the product was extracted into Et_2O (3 × 10 mL). The combined organic phase was washed with brine (10 mL) and dried ($MgSO_4$), and the solvent was removed under reduced pressure. ^{19}F NMR spectrum indicated **9d**:**10d** ratio of 1:9. Purification by column chromatography using silica gel 60 gave **10d** as a white solid (46 mg, 51%); mp 76.0–78.0 °C; R_f 0.34 (hexane–CH_2Cl_2, 80:20); IR (KBr) ν_{max} (cm^{-1}) 3070, 3035, 3001, 2930, 2853, 1613, 1585, 1566, 1498, 1422, 1412, 1299, 1222, 1177, 834, 774; ^1H NMR (500 MHz, $CDCl_3$) δ 2.79–2.84 (m, 2H), 2.86–2.91 (m, 2H), 3.88 (s, 3H), 6.85 (dd, 1H, $^3J_{HH}$ = 8.3 Hz, $^4J_{HH}$ = 2.6 Hz), 7.18 (ddd, 1H, $^3J_{HH}$ = 8.3 Hz, $^4J_{HH}$ = 1.0 Hz, $^5J_{HH}$ = 0.7 Hz), 7.26 (d, 1H, $^4J_{HH}$ = 2.6 Hz), 7.31 (br d, 1H, $^3J_{HH}$ = 8.3 Hz), 7.59 (dd, 1H, $^3J_{HH}$ = 8.3 Hz, $^4J_{HH}$ = 2.3 Hz), 8.05 (d, 1H, $^4J_{HH}$ = 2.2 Hz); ^{13}C NMR (126 MHz, $CDCl_3$) δ 27.5, 29.1, 55.5, 109.8, 113.6, 121.2 (quin, $^3J_{CF}$ = 4.5 Hz), 124.6 (quin, $^3J_{CF}$ = 4.4 Hz), 128.3, 129.2, 129.5, 133.9, 135.2, 141.3, 153.0 (quin, $^2J_{CF}$ = 16.9 Hz), 159.0; ^{19}F NMR ($CDCl_3$) δ 62.5 (d, 4F, $^2J_{FF}$ = 149.9 Hz), 84.1–85.1 (m, 1F); MS (EI) *m*/*z* (rel. int.) 337 (18), 336 (100) $[M]^+$, 208 (54), 165 (44); HRMS (EI) *m*/*z* calcd for $C_{15}H_{13}F_5OS$ $[M]^+$ 336.0607; found, 336.0606.

6. Synthesis of 3-methoxy-6-(pentafluorosulfanyl)phenanthrene (11d). To a stirred solution **10d** (34 mg, 0.10 mmol) in MeOH (1mL) was added CAN (139 mg, 0.25 mmol, 2.5 equiv). The resulting solution was stirred at rt for 16 h, the solvent was removed under reduced pressure and the residue was purified by silica gel column chromatography to give **11d** as a white solid (26 mg, 77%); mp 93.0–95.0 °C; R_f 0.35 (hexane–CH_2Cl_2, 80:20); IR (KBr) ν_{max} (cm^{-1}) 2836, 2838, 1621, 1514, 1422, 1228, 924, 825, 772; ^1H NMR (400 MHz, $CDCl_3$) δ 4.03 (s, 3H), 7.28 (dd, 1H, $^3J_{HH}$ = 8.7 Hz, $^4J_{HH}$ = 2.4 Hz), 7.57 (d, 1H, $^3J_{HH}$ = 8.6 Hz), 7.76 (d, 1H, $^3J_{HH}$ = 8.8 Hz), 7.81 (d, 1H, $^3J_{HH}$ = 8.8 Hz), 7.87–7.89 (m, 2H), 7.91 (d, 1H, $^4J_{HH}$ = 2.5 Hz), 8.89–8.95 (m, 1H); ^{13}C NMR (100 MHz, $CDCl_3$) δ 55.6, 117.6, 120.9 (quin, $^3J_{CF}$ = 4.9 Hz), 123.1–123.3 (m), 126.9, 128.8, 128.9, 129.5, 130.3, 131.5, 133.7, 151.1–151.9 (m), 159.1; ^{19}F NMR (377 MHz, $CDCl_3$) δ 63.5 (d, 4F, $^2J_{FF}$ = 150.3 Hz), 83.9–85.8 (m, 1F); MS (EI) *m*/*z* (rel. int.) 335 (18), 334 (100) $[M]^+$, 291 (21), 183 (28); HRMS (EI) *m*/*z* calcd for $C_{15}H_{11}F_5OS$ $[M]^+$ 334.0451; found, 334.0458.

7. Synthesis of diethyl 4-(pentafluorosulfanyl)benzylphosphonate (12). A suspension of Raney nickel (100 mg) in water was washed with ethanol (2 × 10 mL). A solution of **4** (390 mg, 0.98 mmol) in EtOH (10 mL) was added and the mixture was hydrogenated at 1 atm of H_2 for 3.5 h, followed by filtration, washing with THF (3 × 10 mL), and concentration of the filtrate under reduced pressure. The resulting residue (391 mg, 91%) was used without further purification. Part of the residue (116 mg, 0.314 mmol) was added to concentrated HCl (2 g, 19.2 mmol, 63 equiv), the mixture was cooled to 0 °C, and a solution of $NaNO_2$ (108 mg, 1.57 mmol, 5 equiv) in water (0.2 mL) was added. After 40 min of stirring at 0 °C, 50% aqueous solution of H_3PO_2 (1.71 g, 13.0 mmol, 41 equiv) was added and the mixture was slowly heated to rt. After 20 min at rt, water (5 mL) was added and the product was extracted into Et_2O (3 × 10 mL) and dried ($MgSO_4$), and the solvent was removed under reduced pressure. Purification by column chromatography using silica gel 60 (hexane–acetone, 50:50) gave **12** (97 mg, 87%, 79% from **4**) as a pale brown solid; mp 75–79 °C; R_f 0.62 (hexane–acetone, 50:50); IR (KBr) ν_{max} (cm^{-1}) 3107, 3079, 3060, 3042, 2987, 2933, 2911, 2873, 1602, 1499, 1248, 1055, 1029, 969, 847; ^{1}H NMR (500 MHz, $CDCl_3$) δ 1.27 (td, 6H, $^3J_{HH}$= 7.1 Hz, $^4J_{HP}$ = 0.5 Hz), 3.19 (d, 2H, $^2J_{HP}$ = 22.1 Hz), 4.02–4.09 (m, 4H), 7.40 (dd, 2H, $^3J_{HH}$ = 8.2 Hz, $^4J_{HP}$ = 1.7 Hz), 7.68–7.72 (m, 2H); ^{13}C NMR (126 MHz, $CDCl_3$) δ 16.3 (d, $^3J_{CP}$ = 6.0 Hz), 33.5 (d, $^1J_{CP}$ = 138.2 Hz), 62.3 (d, $^2J_{CP}$ = 6.9 Hz), 125.9–126.3 (m), 129.9 (d, $^3J_{CP}$ = 6.4 Hz), 136.0 (d, $^2J_{CP}$ = 9.2 Hz), 152.60 (quind, $^2J_{CF}$ = 18.3, $^5J_{CP}$ = 3.9 Hz); ^{19}F NMR (377 Hz, $CDCl_3$) δ 62.5 (d, 4F, $^2J_{FF}$ = 150.3 Hz), 83.2–85.0 (m, 1F); ^{31}P NMR (162 MHz, $CDCl_3$) δ 25.3; MS (EI) *m/z* (rel. int.) 354 (8) $[M]^+$, 298 (27), 217 (39), 109 (100), 90 (92), 81 (38); HRMS (ESI^-) *m/z* calcd for $C_{11}H_{17}F_5O_3PS$ $[M–H]^-$ 355.05507; found, 355.05509.

8. Synthesis of (*E*)-1-methoxy-4-(4-(pentafluorosulfanyl)styryl)benzene (13d). A solution of **12** (50 mg, 0.15 mmol) and *p*-methoxybenzaldehyde (18 µL, 0.15 mmol, 1 equiv) in MeCN (0.25 mL) was added to a mixture of KOH (25 mg, 0.45 mmol, 1.8 equiv) in MeCN (2.5 mL) and water (21 µL, 8 equiv). The resulting mixture was stirred at rt for 1 h, followed by the addition of water (5 mL) and removal of MeCN under reduced pressure. The product was extracted into Et_2O (3 × 8 mL), the combined organic phase was washed with water (8 mL) and brine (8 mL), and dried ($MgSO_4$), and the solvent was removed under reduced pressure. Column chromatography using silica gel 60 (hexane–EtOAc, 92:8) gave **13d** as a white solid (36 mg, 72%); mp 129.8–132.5 °C (hexane); R_f 0.38 (hexane–EtOAc, 95:5); IR (KBr) ν_{max} (cm^{-1}) 3097, 3042, 3017, 2980, 2920, 2850, 1606, 1593, 1569, 1516, 828;

^{1}H NMR (500 MHz, $CDCl_3$) δ 3.83 (s, 3H), 6.89–6.92 (m, 2H), 6.94 (d, 1H, $^{3}J_{HH}$ = 16.2 Hz), 7.12 (d, 1H, $^{3}J_{HH}$ = 16.3 Hz), 7.44–7.48 (m, 2H), 7.50 (br d, 2H, $^{3}J_{HH}$ = 8.5 Hz), 7.67–7.72 (m, 2H); ^{13}C NMR (126 MHz, $CDCl_3$) δ 55.3, 114.3, 124.2, 126.0, 126.3 (quin, $^{3}J_{CF}$ = 4.6 Hz), 128.2, 129.2, 131.5, 141.0, 152.2 (quin, $^{2}J_{CF}$ = 17.4 Hz), 160.0; ^{19}F NMR (470 MHz, $CDCl_3$) δ 62.6 (d, 4F, $^{2}J_{FF}$ = 150.0 Hz), 84.0–85.4 (m, 1F); MS (EI) *m*/*z* (rel. int.) 337 (17), 336 (100) $[M]^+$, 166 (47), 165 (49); HRMS (EI) *m*/*z* calcd for $C_{15}H_{13}F_5O_2S$ $[M]^+$ 336.0607; found, 336.0602.
